# Evidences of Herbal Medicine-Derived Natural Products Effects in Inflammatory Lung Diseases

**DOI:** 10.1155/2016/2348968

**Published:** 2016-06-29

**Authors:** Fernanda Paula R. Santana, Nathalia M. Pinheiro, Márcia Isabel B. Mernak, Renato F. Righetti, Mílton A. Martins, João H. G. Lago, Fernanda D. T. Q. dos Santos Lopes, Iolanda F. L. C. Tibério, Carla M. Prado

**Affiliations:** ^1^Instituto de Ciências Ambientais, Químicas e Farmacêuticas, Federal University of São Paulo, Diadema–SP 09972-270, Brazil; ^2^School of Medicine, University of São Paulo, São Paulo–SP 01246903, Brazil; ^3^Instituto de Saúde e Sociedade, Federal University of São Paulo, Santos–SP 11015-020, Brazil

## Abstract

Pulmonary inflammation is a hallmark of many respiratory diseases such as asthma, chronic obstructive pulmonary disease (COPD), and acute respiratory syndrome distress (ARDS). Most of these diseases are treated with anti-inflammatory therapy in order to prevent or to reduce the pulmonary inflammation. Herbal medicine-derived natural products have been used in folk medicine and scientific studies to evaluate the value of these compounds have grown in recent years. Many substances derived from plants have the biological effects* in vitro* and* in vivo*, such as flavonoids, alkaloids, and terpenoids. Among the biological activities of natural products derived from plants can be pointed out the anti-inflammatory, antiviral, antiplatelet, antitumor anti-allergic activities, and antioxidant. Although many reports have evaluated the effects of these compounds in experimental models, studies evaluating clinical trials are scarce in the literature. This review aims to emphasize the effects of these different natural products in pulmonary diseases in experimental models and in humans and pointing out some possible mechanisms of action.

## 1. Introduction

Pulmonary diseases rate has been increasing and it is related to causes of death for decades. Among the pulmonary diseases, chronic obstructive pulmonary diseases (COPD), asthma, and acute respiratory distress syndrome (ARDS) are the most common and are linked to high mortality and/or high morbidity rates [1–3]. These pulmonary diseases have in common the pulmonary inflammation which could be acute or chronic and the orchestration of a lot of inflammatory mediators [4].

Inflammation is a cellular response that can occur in the lung induced by external or internal agents. Although it is an important response in the organism, the chronic inflammation could damage the lung. Lung inflammation involves the activation of inflammatory cells, such as macrophages, lymphocytes, neutrophils, and eosinophils, which is a source of different kinds of inflammatory mediators such as histamine, tumor necrosis factor (TNF-*α*), interleukins, IL-1*β*, IL-4, IL-5, and IL-6, prostaglandins, leukotrienes, and nitric oxide. The release of these inflammatory mediators is related to the signals and symptoms observed in pulmonary diseases such as loss of lung function, airway hyperresponsiveness and obstruction, airway edema, mucus hypersecretion, and lung remodeling [3, 4].

The treatment of the respiratory disorders often involves the use of anti-inflammatory therapies and it is required not only to counteract lung inflammation but also to avoid the remodeling process and the destruction of lung tissue preventing a decrement in lung function that is observed in many patients [5]. Additionally, pulmonary diseases such as emphysema and ARDS do not have proper treatments yet, requiring more studies in alternative therapy [6, 7].

**Figure 1:**
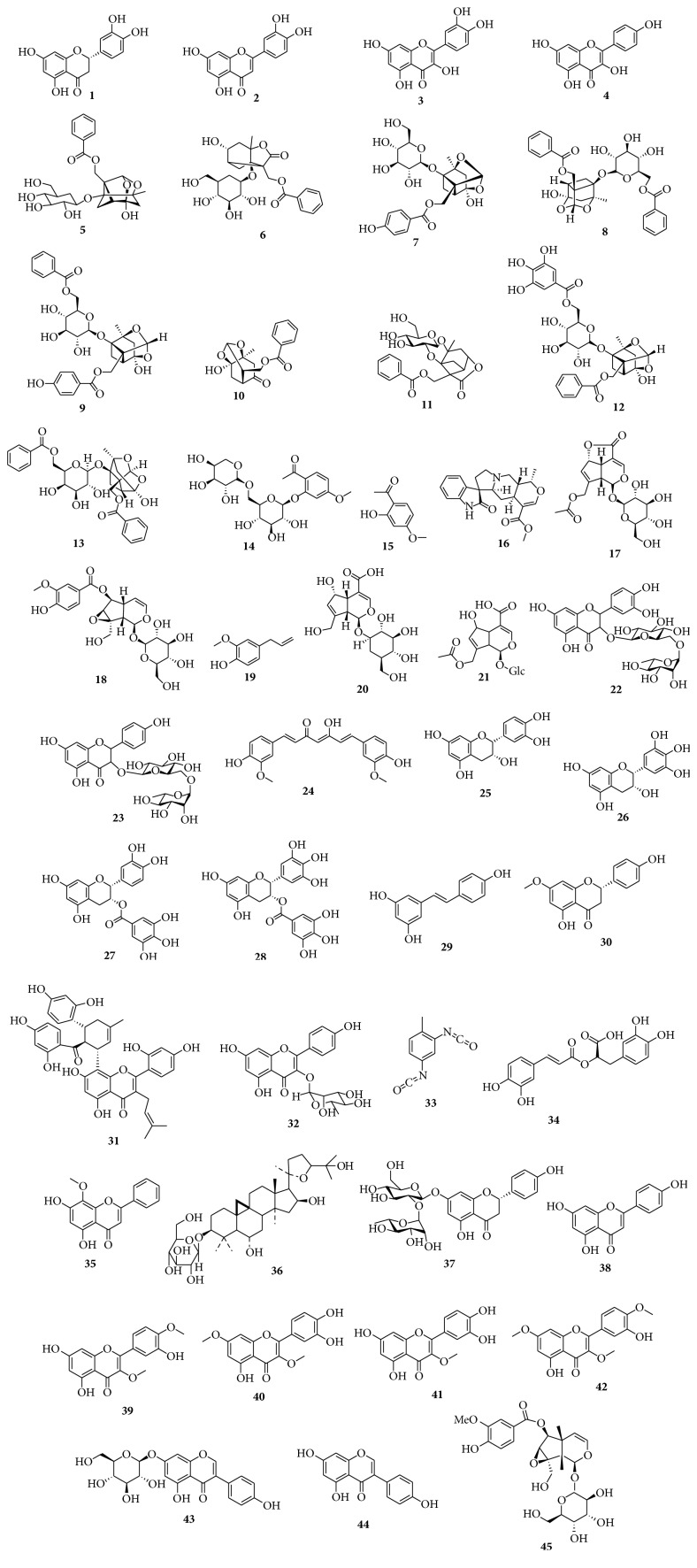
Natural products (**1**–**45**) from plants with effects in inflammatory lung diseases.

Herbs have been used in folk medicine since many years and the use of herbal-derived natural products as a therapeutic tool has been increasing considerably [8–10]. However, several herbal-derived natural compounds significantly affect cellular mechanisms and evidence of the benefic effects of herbal-derived natural products in inflammatory pulmonary diseases has been increasing [8]. The use of these compounds must be based on scientific evidence. The structures of the various products cited in this review are presented in Figure 1. Herein, we review the anti-inflammatory activity of herbal-derived natural products with respect to three pulmonary diseases, namely, asthma, emphysema, and ARDS (Table 1).

## 2. Evidence of Herbal-Derived Natural Products in ARDS

ARDS is characterized by acute pulmonary inflammation primary characterized by neutrophil infiltration, interstitial edema, and hypoxemia, is often accompanied by aggressive fibrosis, and is still one of the leading causes of death within an intensive care unit [11–13].

The acute phase of ARDS is characterized by local inflammation and systemic response [14]. Experimental and clinical studies have sought to elucidate the complex mechanisms involved in the pathogenesis of secondary injury. Several mechanisms remain little understood; however, knowing the functions of cytokines and inflammatory mediators involved in the process helps us to clarify the process of injury and repair in ARDS.

Some specific cells are able to secrete some soluble protein called cytokines, which have the ability to modify the behavior of other cells [15]. TNF-*α* and interleukin 1 beta and 8 (IL-1 *β* and IL-8) [15], cytokines classified as proinflammatory [14], are included among the cytokines involved in acute phase of ARDS.

Patients with ARDS more than 72 hours usually evolve into the late phase of ARDS which is characterized by having diffuse alveolar damage that is sometimes irreversible [16]. Although mortality has been reduced due to the improved control of ventilation, no effective pharmacological treatment for the disease is available until now.

Many experimental studies seek evidence in natural substances to help in treating this disease; however, no evidence of the use of herbal medicine in patients with ARDS was found. Several studies demonstrated that plants can have effects on cell migration, anti-inflammatory, cytokines, metalloproteinase, oxidative stress, and the downregulation of several transcription factors making as a potential therapeutic use in ARDS. Currently, the phytochemical groups surveyed more with anti-inflammatory and antimicrobial actions are the flavonoids, alkaloids, and glycosides [17–19].

Eriodictyol (**1**), a flavonoid isolated from the Chinese herb* Dracocephalum rupestre*, has long been established as an antioxidant and anti-inflammatory agent. Zhu et al. [20] investigated the effects of eriodictyol on lipopolysaccharide- (LPS-) induced acute lung injury (ALI) in mice and have demonstrated that eriodictyol alleviates the LPS-induced lung injury in mice by regulating the transcription factor nuclear factor erythroid-2-related factor 2 (Nrf2) pathway and inhibiting the expression of inflammatory cytokines in macrophages.

Kuo et al. [21] showed that pretreatment with flavonoids luteolin (**2**) reduced pulmonary hemorrhage and neutrophilic inflammation, as well as interstitial edema. The control of pulmonary inflammation was due to the reduction of cytokines such as TNF-*α*, KC, and ICAM-1 content in the bronchoalveolar lavage fluid (BALF). The control of oxidative damage and lipid peroxidation was attributed to reduced activity of catalase and superoxide dismutase activity. The mechanism involved is related to the effects of luteolin in the inhibition of NF-*κ*B and the MAPK activity.

Quercetin (**3**) is also evaluated in a LPS-induced experimental ALI and it reduced the release of proinflammatory cytokines in the BALF, such as TNF-*α*, IL1-*β*, and IL-6, by a heme oxygenase-1(HO-1) dependent pathway [22]. Other authors also demonstrated that quercetin is effective in reducing serum cytokines TNF-*α*, IL1-*β*, IL-6, and nitric oxide (NO) and the mechanism involves an increase in IL-10 secretion, an anti-inflammatory cytokine [23]. In addition to these cytoprotective effects, quercetin also decreased the ratio of lung weight to body weight and MMP-9 activity [22, 23]. Quercetin also reduced lung permeability, the number of macrophages and neutrophils, and the myeloperoxidase activity. Wang et al. [23] also showed that quercetin is effective in reducing COX-2, iNOS expression, HMGB1, and p65-nuclear factor kappa B (NF-*κ*B) [23].

Kaempferol (**4**), naturally occurring flavonoid, when evaluated in ALI models induced by LPS appeared effective in reducing pulmonary edema as well as the bleeding and thickness of the alveolar wall. The kaempferol also reduced the inflammatory cells and total protein in the BALF and cytokines including TNF-*α*, IL-1*β*, and IL-6. Despite the increase in superoxide dismutase activity, the mechanism of action is through the control of signaling pathways MAPK and NF-*κ*B [24].

The dried root* Paeonia lactiflora* has been used as a medicinal herb in traditional Chinese medicine for centuries. The glucosides of peony (TGP), the water/ethanol extract of the dried* Paeonia lactiflora*, contain more than 15 components, including paeoniflorin (**5**), albiflorin (**6**), oxypaeoniflorin (**7**), benzoylpaeoniflorin (**8**), oxybenzoyl-paeoniflorin (**9**), paeoniflorigenone (**10**), lactiflorin (**11**), galloylpaeoniflorin (**12**), paeonin (**13**), paeonolide (**14**), and paeonol (**15**). Using a model of LPS-induced ALI, He and Dai [25] demonstrated that TGP markedly suppressed LPS-induced NO production and inducible nitric oxide synthase (iNOS) expression peritoneal macrophages from rats. In addition, the production of reactive oxygen species from LPS-stimulated macrophages was inhibited. Kim and Ha [26] showed that paeoniflorin (**5**), the principle component of TGP, was effective in inhibiting the NO and PGE2 production induced by LPS in stimulated macrophages. Further experiments showed that paeoniflorin inhibited LPS-stimulated TNF-*α* and interleukin- (IL-) 1*β* release and promoted an increase in IL-10 production [27].

Mitraphylline (**16**) is the major pentacyclic oxindolic alkaloid presented in* Uncaria tomentosa* and has traditionally been used to treat inflammatory diseases [28]. However, the specific role of mitraphylline in inflammation is still not clear. Some studies provided its ability to inhibit proinflammatory cytokines, such as TNF-*α*, through NF-*κ*B-dependent mechanism. TNF-*α* primes neutrophils and modulates phagocytic and oxidative burst activities in inflammatory processes [29]. Recently, Montserrat-de la Paz et al. [30] investigated the effects on mitraphylline in LPS-activated human primary neutrophils including activation of surface markers by FACS and the expression of inflammatory cytokines. The treatment with mitraphylline reduced the activated neutrophils CD16(+)CD62L(−) and the expression and secretion of proinflammatory cytokines (TNF-*α*, IL-6, or IL-8) to the levels obtained in basal control condition.

Asperuloside (**17**), an iridoid glycoside found in* Herba Paederiae*, is a product from traditional Chinese herbal medicine. Qiu et al. [31] investigated the protective effects of asperuloside on inflammatory responses in a LPS-induced ALI model. This compound was able to downregulate TNF-*α*, IL-1*β*, and IL-6 levels both* in vitro* and* in vivo*. Additionally, the treatment with asperuloside also reduced the lung wet-to-dry weight, histological alterations, and myeloperoxidase activity in this model. The mechanism involved in the effects of asperuloside is related to the phosphorylation of the inhibitor of NF-*κ*B (I*κ*B*α*), extracellular signal-related kinases 1 and 2 (ERK1/2), and c-Jun N-terminal kinase (JNK) and p38 mitogen-activated protein kinase (p38MAPK) in LPS-induced lung inflammation. These results indicate that asperuloside exerts its anti-inflammatory effect in correlation with the inhibition of proinflammatory mediators through suppressing NF-*κ*B translocation and MAPK phosphorylation.

The rhizome of* Picrorhiza scrophulariiflora* has been described as part of Asian traditional medicine for the treatment of rather a broad range of diseases [32], including tumors and liver infections. Picroside II (**18**) is known as a major constituent of this plant [33] and it was reported that the herb has immunomodulatory and anti-inflammatory functions. The ethanol extract of* P. scrophulariiflora* suppresses redox-sensitive inflammation [34], while crude extract of* P. scrophulariiflora* reduces the classical pathway of complement activation, the production of ROS by activated neutrophils, and the proliferation of T lymphocytes [35]. Noh et al. [36] using a model of RAW 264.7 cells and also an* in vivo* model of LPS-induced ALI showed that Picroside II was effective in suppressing neutrophilic lung inflammation and that the possible anti-inflammatory effect of Picroside II was, at least in part, associated with TGF-*β* signaling.

The phenylpropanoid eugenol (**19**) is a substance present in essential oils of various plants, and it is part of a phenolic group with a recognized antioxidant capacity [37]. Eugenol can prevent lipid peroxidation [38] and also inhibit the formation of superoxide radicals from xanthine oxidase system and the generation of hydroxyl radical [39, 40]. The relationship between oxidative stress and inflammation in ARDS is not completely understood; however, it is fact that the reduction of oxidative stress culminates with reduction in inflammatory mediators release by inflammatory cells [41]. Murakami et al. [42] showed that eugenol inhibits the transcription of NF-*κ*B and COX-2 stimulated by LPS. According with these findings, Huang et al. [43] showed that animals which received eugenol have reduction in proinflammatory cytokines and inflammatory cells in BALF due to an antioxidative effect of this product and by inhibition of the transcription of NF-*κ*B in lung homogenate.

As reported in the literature [44, 45], the crude extracts from fruits and leaves of* Morinda citrifolia* have been used in traditional medicine for the treatment of inflammation and pulmonary diseases. These effects could be associated with the presence of several antioxidant natural products such as iridoid glycosides (deacetylasperulosidic acid,** 20** and asperulosidic acid,** 21**), and flavonoids (quercetin-3-O-*α*-L-rhamnopyranosyl-(1→6)-*β*-D-glucopyranoside,** 22** and kaempferol-3-O-*α*-L-rhamnopyranosyl-(1→6)-*β*-D-glucopyranoside,** 23**). Additionally, turmeric (*Curcuma longa*) and ginger (*Zingiber officinale*) displayed an important effect in inflammation, including lung and pulmonary, which could be due the presence of curcumin (**24**) [46, 47].

The presence of different polyphenol derivatives in green tea (*Camellia sinensis* leaves) such as catechin (epicatechin,** 25**; epigallocatechin,** 26**; epicatechin gallate,** 27;** and epigallocatechin gallate,** 28**) is associated with the potent chemopreventive agent against lung cancer formation in animal studies [48]. The reported mechanisms for activity of green tea against cancer are antioxidation, induction of phase II enzymes, inhibition of TNF-*α* expression and release, inhibition of cell proliferation, and induction of apoptosis.

Finally, considering that antioxidant derivatives could be associated with anti-inflammatory effects and, consequently, with the lung diseases treatment, the red wine could be considered an important source of bioactive compounds due to accumulation of stilbenes, especially resveratrol (**29**). As reported [49, 50], treatment using resveratrol inhibits lung cancer cell growth via induction of premature senescence through ROS-mediated DNA damage.

Many other natural compounds have been effective in the reduction of proinflammatory cytokines both in BALF, in the lung tissue, and in serum [51–53]. Despite evidence showing that many natural substances were effective in controlling the alterations observed in models of in ALI, there is no evidence, at least in our knowledge, related to the effects of herbal medicine in ARDS in humans. There is still need for more studies to better understanding the mechanisms involved and to elucidate the efficacy and the safety of natural compounds to a subsequent clinical application in patients.

## 3. Evidence of Herbal-Derived Natural Products in Bronchial Asthma

Asthma is a heterogeneous and complex chronic respiratory disease associated with several phenotypes. Generally, the symptoms of asthma are recurrent episodes of wheezing, breathlessness, chest tightness, and coughing particularly at night or early in the morning that are typically associated with variable airflow obstruction [1]. Approximately 10% of the world population is affected by the disease, varying from 1 to 18%. Clinically the patients present symptoms such as wheeze, dyspnea, chest tightness, and cough that vary over time and in intensity, which is associated with airflow limitation [1].

Asthma depends on the interaction of genetic and environmental factors featured by the activation of the Th2 profile cells, eosinophils, mast cells, and neutrophils [54]. Several mediators/modulators are involved in asthma pathogenesis and in hyperresponsiveness such as Th2 cytokines (IL-5, IL-4, and IL-13), IL-17, leukotrienes, nitric oxide, Rho kinase, cholinergic system, and others that were clearly evaluated in humans and animal models [1, 55–59].

The long-term goals for asthma treatment are to achieve control of symptoms with low side effects of the medications and reduce the risk of exacerbations and of fixed airflow obstruction frequently associated with the remodeling process. Corticosteroids are considered the gold-standard treatment for asthma, including during an acute asthma attack. The corticosteroids are responsible for inhibiting numerous anti-inflammatory pathways. Inhaled or oral corticosteroids are widely used, acting as a powerful anti-inflammatory drug.

Some patients (10 to 25%) remain symptomatic despite optimal glucocorticosteroid therapy and there is evidence of persistent airway and distal lung inflammation and suffering from the unwanted effects of high-prolonged corticoid therapy, such as osteoporosis, cataracts, and diabetes. In addition, corticosteroids do not act directly on pulmonary structural changes better regulating the different processes involved in the pathophysiology of asthma [1, 60, 61]. However, the discovery of new drugs for the control of the disease is essential in patients with severe corticosteroid-insensitive asthma and also for the decrease of the collateral systemic effects of steroid use.

Several studies demonstrated anti-inflammatory effects of flavonoids of different plants in experimental asthma treatment. Toledo et al. [62] investigated the effects of sakuranetin (**30**), a flavonoid from* Baccharis retusa*, in an asthma model and found that this flavonoid induces a reduction of the Th2 cytokines such as IL-5, RANTES, and Eotaxin in sensitized mice. Sakuranetin also reduced the number of inflammatory cells in the lung, particularly eosinophils, and IgE title in the animals sensitized with ovalbumin and treated with this flavonoid. The effects of sakuranetin seem to be related to the inhibition of NF-*κ*B in lung. Reinforcing the effects of flavonoids in asthma, Jung et al. [63] demonstrated that kuwanon G (**31**), a flavonoid isolated from root bark of* Morus alba* L., decreases the levels of IgE in ovalbumin-induced allergic asthma in mice. Further, considering the inflammatory mediators involved in asthma, kuwanon G significantly decreased the levels of IL-4, IL-5, and IL-13 in the sera and in bronchoalveolar lavage of asthma mice. Chung et al. [64] demonstrated using flavonoids kaempferol-3-O-rhamnoside (**32**) and kaempferol (**4**) a reduction in inflammatory cell numbers in BALF and also an inhibition in the increase in Th2 cytokines (IL-4, IL-5, and IL-13) and TNF-*α* protein levels.

The effects of herbal-derived natural compounds on eosinophilic infiltration in different murine models of asthma have been previously demonstrated. Using a methanol extract from* Boerhavia procumbens*, Bokhari and Khan [65] demonstrated a reduction in the infiltration of eosinophils and lymphocytes in lungs of toluene diisocyanate (**33**) exposure in rats. Costa et al. [66] found that* Ocimum gratissimum*, a plant rich in rosmarinic acid (**34**) and commonly used in folk medicine in Brazil, attenuates the eosinophilic airway inflammation and reduced mucus production and IL-4 expression in an experimental model of respiratory allergy to* Blomia tropicalis*. The administration of flavonoid quercetin (**3**) in ovalbumin immunized mice, suggested a reduction in the number of eosinophil in the BALF, neutrophil in peripheral blood, and the levels of IL-5 in lung homogenate [67].

The administration of wogonin (**35**), a flavone derived from a plant called* Scutellariae radix*, reduced the total IgE and ovalbumin specific IgE levels compared with the ovalbumin challenged group [68].* Astragalus membranaceus*, a traditional Chinese herb, decreases airway allergic responses, including specific IgE, eosinophilia, lymphocyte infiltration, and airway inflammation, modulates Th1/2 immune balance, and activates PPAR in a murine asthma model [69]. This plant is composed essentially by saponins (huangqiyiesaponin C,** 36**) and flavonoids (quercetin,** 3** and kaempferol,** 4**), which are known anti-inflammatory metabolites.

Naringin (**37**), a component isolated from the dried unripe or ripe fruit peel of* Citrus grandis *“tomentosa” (*Exocarpium Citri Grandis*), reduced enhanced cough and airway hyperresponsiveness and inhibited the increases in the leukocytes, IL-4, IL-5, and IL-13, in BALF in a model of asthma using guinea pigs [70]. Further, using the same component, Guihua et al. [71] also demonstrated an inhibition of ovalbumin-induced increased airway resistance (Raw) and eosinophil infiltration, as well as IL-4 and INF-*δ* levels after naringin administration. The apigen (**38**), a related flavonoid, also inhibited OVA-induced increases in Raw and eosinophil infiltration in lung tissue associated with a reduction in IL-6, TNF-*α*, and IL-17A levels [72].

Curcumin (**24**), a natural product isolated from the plant* Curcuma longa*, improved the airway inflammation and reversed the increased levels of Notch signaling pathway (Notch1/2) receptors and the transcription factor GATA3 in ovalbumin-sensitized mice [73]. Other studies with intranasal curcumin administration demonstrated a protective effect on the recruitment of inflammatory cells to the airways and in the remodeling features such as peribronchial and airway smooth muscle thickening and mucus secretion in ovalbumin-induced chronic asthma in a murine model [74].

de Oliveira et al. [75] demonstrated the anti-inflammatory effects of* Punica granatum* which inhibited leukocytes recruitment in BALF, especially eosinophils, and decreased cytokines (IL-1*β* and IL-5) release in the lungs of OVA-sensitized BALB/c mice. This effect could be associated with the presence of different metabolites such as alkaloids, terpenoids, and flavonoids.

The remodeling process in asthmatics induces significant structural changes in proximal and distal airways as well as in the distal lung. This process is associated with airway smooth muscle hypertrophy and hyperplasia, mucous gland hyperplasia, and increased thickness of the airway wall [54, 76, 77]. It is well known that inflammatory mediators such as IL-4 and IL-13 as well as the persistence of chronic inflammation in airways are involved in the remodeling process, so natural compounds that inhibit the inflammatory process in asthma may also prevent the remodeling process.

Extracts of* Siegesbeckia glabrescens*, a traditional medicinal plant in Korea, reduced mucus overproduction in airways in a asthma murine model, decreased the expression of iNOS and COX-2, reducing the number of inflammatory cells in BALF and the cytokine release (IL-4, IL-5 and IL-13) [78]. Using an inhalator approach, the treatment with Lavender essential oil (Lvn) in a murine model of bronchial asthma reduced the airway resistance (Raw) and the number of eosinophils recovered in BALF and also in peribronchial and perivascular tissues. Furthermore, Lvn reduced mucous cell hyperplasia and the mRNA expression of Muc5b without significantly changing the mRNA expression of Muc5ac [79]. Chemically, this plant showed to be composed by quercetin flavonoid derivatives such as 3,4′-O-dimethylquercetin (**39**), 3,7-O-dimethylquercetin (**40**), 3-O-methylquercetin (**41**), and 3,7,4′-O-trimethylquercetin (**42**) [80].

Wang et al. [81] contribute to the mechanisms involved in the anti-inflammatory effects of* Yupingfeng Pulvis* in an asthma model, which is related to a reduction of the proportion of Th17 cells, whereas the population of Treg cells in BALF was increased, inhibiting the pulmonary proinflammatory cytokines release in treated animals.

Several studies have associated the diet with the development of allergic diseases. In this context, low consumption of fruits and vegetables rich in antioxidant substances is associated with high risk of asthma and atopic diseases development. However, the diet rich in antioxidants and lipids particularly during pregnancy and childhood is related to reducing the allergic diseases prevalence [82]. In a population-based study, the asthma incidence was lower at higher quercetin (**3**) and naringin (**37**) flavonoids intake from the diet [83]. However, Smith et al. [35] showed that the consumption of soy isoflavone such as genistin (**43**) and genistein (**44**) did not improves asthma control in adolescents and adults patients with poorly controlled asthma.

Studying asthmatic patients, Watson et al. [84] investigated the effects of the purple passion fruit peel (PFP) extract and observed that patients who received oral administration of purple passion fruit peel showed a reduction of wheeze and cough as well as shortness of breath. An herbal known as Umckaloabo® with contains root extract of* Pelargonium sidoides* seems to be effective in the treatment of acute respiratory infections [85, 86]. Additionally, Tahan and Yaman [87] demonstrated that Umckaloabo reduced asthma attack during upper respiratory tract viral infections in asthmatic children which demonstrated a minor cough frequency and nasal congestion. Collectively, these data suggest that high intake of fruit and vegetables had a negative association with risk of asthma.

## 4. Evidence of Herbal-Derived Natural Products in COPD

Chronic obstructive pulmonary disease (COPD) is the major cause of chronic respiratory morbidity and mortality and the fifth cause of death worldwide [91, 92] and it is characterized by a persistent airflow limitation associated with an enhanced chronic inflammatory response to noxious particles or gases [92]. This inflammatory response usually promotes parenchymal destruction (resulting in emphysema) and small airways fibrosis [92]. The incidence and severity of chronic obstructive pulmonary disease (COPD) are growing affecting between 100 and 150 million people worldwide [92].

COPD is characterized by persistent airflow limitation that is usually progressive and is associated with an enhanced chronic inflammatory response to noxious particles or gases in the airways and the lung [92]. The inhalation of cigarette smoke and other environmental pollutants stimulates the alveolar macrophages in the lung epithelial cells to generate reactive oxygen species and reactive nitrogen species [93] in excess causing an imbalance in the system.

The protease/antiprotease imbalance is still recognized as the main mechanism enrolled in emphysema development [94–97]. The majority of studies in animal models have attested the importance of MMPs in parenchymal destruction in emphysema [98–100], especially the MMP-12; other proteases such as neutrophil elastase from the serine proteases family are described in humans emphysema [101].

These proteases are produced by neutrophils, macrophages, and bronchial epithelial cells [102–104]. In emphysema, there is an increase in neutrophils in sputum and airways, while macrophages are predominate in parenchyma and in bronchoalveolar lavage, suggesting an importance of these cells in distal airways [105, 106]. In this context, elastase secreted by neutrophils and macrophages may play an important role in lung tissue destruction [107, 108].

The proteolytic attack of extracellular matrix components of lung parenchyma by proteases induces alveolar walls destruction associated with a dynamic tissue repair and remodeling process, involving a reorganization of extracellular matrix (ECM) components [109]. However, biochemical and histological studies have demonstrated that the remodeling of col I and III, elastin, and fibrillin results in loss of lung elasticity [110–112].

The COPD management is divided into some approaches such as the reduction of risk factors and therapeutic management of stable disease and of the exacerbations. However, no currently available treatments reduce the progression or even adequately suppress the inflammation in small airways and lung parenchyma [92]. Therefore, we have to consider the importance of new approaches in emphysema studies, especially in concern with the new targets to avoid the progression of the alveolar walls destruction and remodeling.

There are a large number of protein families that are able to inhibit serine, cysteine, and metalloproteases [113], and the mechanisms of inhibition could be attributed to the catalytic mechanisms of proteases action or a mechanism-unrelated blockage of the specific active sites [113].

Animal models of emphysema have been used as features to better clarify the pathogenesis of such disease and the most used are the elastases and cigarette-induced models. However, both of them showed advantages and disadvantages that have been considered for each experimental approach. Although, the CS-induced model is suggested as the best to reproduce the pathogenesis of humans emphysema; the parenchymal destruction is mild even after long time of exposure, whereas the elastase instillations produce severe emphysema, depending on the dose, in short times [114–116], but, so far it is not a model that resembles the physiology of this disease as in humans.

Several antioxidant agents such as thiol molecules, polyphenols derived from natural products, and other substances such as curcumin, resveratrol, andquercetin have been evaluated in models of emphysema. Considering that the physiopathology of COPD involves oxidative stress and protease-antiprotease unbalance, substances that affect these aspects should be considered to be beneficial in COPD. There are studies showing the effects of protease inhibitors against the emphysema development and progression in experimental models [94, 117, 118].

In a previous study, Lourenço et al. [119] demonstrated in elastase-induced model that a treatment with a serine protease inhibitor from the cattle tick (rBmTI-A) attenuated emphysema in treated mice before and after the elastase intranasal instillation. The rBmTI-A treatment was responsible to reverse the loss of elastic recoil, the alveolar enlargement, and the total inflammatory cells amount in bronchoalveolar lavage. Indeed, it was sufficient to reduce the density of positive cells for metalloprotease-12, suggesting that this MMP could be a therapeutic target [119].

Wright et al. [120] showed in guinea pigs acutely or chronically exposed to cigarette smoke that the treatment with a serine elastase inhibitor (ZD0892) decreased the inflammatory activity mediated in part by neutrophils with a reduction in parenchymal destruction [120]. Also, Kuraki et al. [121] performed a prior treatment with an oral inhibitor for neutrophil elastase (ONO-6818) before the induction of acute lung injury and emphysema in rats by intratracheal administration of human neutrophil elastase. They observed that the ONO-6818 treatment inhibited lung hemorrhage and the neutrophils increase at the acute phase and that, in long term, it prevented HNE-induced emphysema.

Takayama et al. [118] performed a posttreatment with a neutrophil elastase inhibitor (NEI; ONO-5046) after intestinal ischemia-reperfusion in rats and observed a reduction in neutrophil activation in pulmonary vessels and neutrophil infiltration in the lungs, preventing the lung injury development.

Considering the herbal medicine, few studies have evaluated the effects of these compounds in emphysema. Moreover, it is difficult to isolate the effects of these compounds in the inhibition of elastase activity or in the inflammatory mediators. In this context, Sartor et al. [122] showed that the flavonoid epigallocatechin gallate (**28**) is an inhibitor of leukocyte elastase. Lee et al. [89] showed that an herbal formula PM014 reduced inflammatory cells in BALF and the levels of TNF-*α* and IL-6 in a model of elastase+LPS-induced emphysema.

More recently, Lee et al. [90] evaluated the effects of* Callicarpa japonica*, a traditionally herbal used in oriental countries to treat inflammatory diseases. The obtained results indicated that* Callicarpa japonica* extracts reduced neutrophil infiltration, the production of cytokines IL-6 and TNF-*α*, and the oxidative stress in a model of cigarette smoke. Additionally, the authors found a reduction in the production of mucus in lung tissue of cigarette smoke animals. The mechanisms involved were evaluated* in vitro* and were related to a reduction in the phosphorylation of ERK.

Other traditional Chinese medicines have been used to treat chronic obstructive pulmonary disease (COPD) in patients [123, 124]. In this context, Li et al. [123] demonstrated that the Bu-Fei Jian-Pi granules, Bu-Fei Yi-Shen granules, and Yi-Qi Zi-Shen granules, three common Chinese medicines, have beneficial effects on measured outcomes in stable COPD patients over the 6-month treatment. Moreover, Li et al. [124] demonstrated the benefic effects of the three Tiao-Bu Fei-Shen (Bu-Fei Jian-Pi, Bu-Fei Yi-Shen, and Yi-Qi Zi-Shen granules) in the reduction in the levels of IL-1*β*, TNF-*α*, p-NF-*κ*B, p-I*κ*B*α*, TGF-*β*1, and Smad2 in COPD rats.

Song et al. [125] studied the Picroside C (**45**), isolated from* Pseudolysimachion rotundum,* and found that this compound protects the neutrophil influx, the production of reactive oxygen species, and the classical cytokines IL-6 and TNF-*α* as well as the elastase activity. The author suggested that the mechanism involved is the inhibition of the NF-*κ*B pathway which counteracts the lung inflammation.

Our group also evaluated the effects of sakuranetin in model of elastase-induced emphysema. We found that sakuranetin (**30**), isolated from* Baccharis retusa*, prevented the alveolar destruction in a model of elastase-induced emphysema. Moreover, the authors showed that this compound reduced lung remodeling and the levels of TNF-*α*, IL-1*β*, and M-CSF in the BALF. The mechanisms involved are associated with the inhibition of NF-*κ*B and oxidative stress in lung [88].

At least, in our knowledge, only the group of Mukaida et al. [126] has evaluated the effects of a natural compound in COPD patients. The authors tested the effects of a mix of six herbal compounds and found that elderly COPD patients have reduced the intensity of the cough; however, other parameters were not evaluated. These findings reinforce the importance of oxidative stress and protease/antiprotease imbalance in emphysema development and suggest oxidative stress, MMP-12, and neutrophil elastase as a future targets for emphysema therapy.

## 5. Conclusions

**Figure 2:**
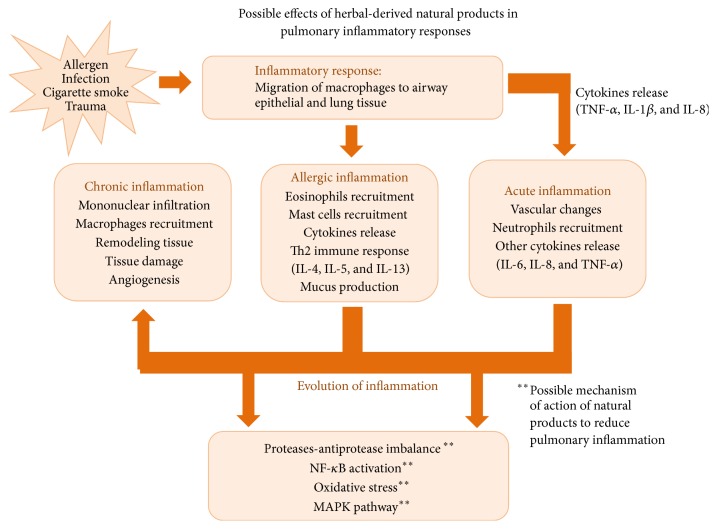
Possible mechanism involved in the effects of herbal-derived natural products on pulmonary inflammation in respiratory diseases. It is believed that the natural products derived from plants inhibit the pulmonary inflammation by the inhibition of the transcription of NF-*κ*B to the nucleus, thus preventing the development of all the inflammatory processes triggered by allergen, cigarette smoke, virus, or bacteria. Therefore, these products can reduce the inflammatory cytokines release and oxidative stress. These effects together culminate with the improvement of lung function and with the reduction of pulmonary inflammation.

In conclusion, herbal medicine-derived natural products can be considered as an alternative therapeutic potential for respiratory diseases since several compounds showed anti-inflammatory effects inhibition different inflammatory mediators involved in respiratory diseases such as asthma, ARDS, and COPD. The possible mechanisms involved in the effects of natural products in pulmonary inflammation and respiratory diseases are summarized in the Figure 2. Most of the studies are pointing out the effects of natural products on the inhibition of NF-*κ*B and MAPK pathways, besides the antioxidant effects associated with these products. However, the clinical trials using these compounds are scarce in the literature and the safety and efficacy should be confirmed for further studies.

## Figures and Tables

**Table 1:** Herbal-derived natural compounds effects in lung inflammation and diseases.

	Inflammatory mediators	Effects in	Reference
*Natural products*			

Eriodictyol (**1**)	Regulation of Nrf2 pathway and inhibiting the expression of inflammatory cytokines TNF-*α*, IL-6, IL-1*β*, and in BALF and serum	ALI model	[20]
Luteolin (**2**)	Reduction of TNF-*α*, KC, ICAM-1, SOD, activations of MAPK, and NF-*κ*B pathways and neutrophils inflammation	ALI model	[21]
Quercetin (**3**)	Reduction of TNF-*α*, IL1-*β*, IL-5, and IL-6 in BALF, NO COX-2, iNOS expression, HMGB1, and p65NF-*κ*B. Increase IL-10 secretion.	ALI model, asthma model	[22, 23, 67]
Kaempferol (**4**)	Reduction of inflammatory cells, activation of MAPK, and NF-*κ*B pathways	ALI model	[24]
Mitraphylline (**16**)	Reduction of IL-1*α*, IL-1*β*, IL-17, TNF-*α*, IL-6, and IL-8.	ALI model	[29, 30]
Asperuloside (**17**)	Reduction of TNF-*α*, IL-1*β*, and IL-6 levels	ALI model	[31]
Eugenol (**19**)	Inhibition of superoxide radicals from xanthine oxidase system and the generation of hydroxyl radical	ALI model	[39]
Sakuranetin (**30**)	Reduction of eosinophils, TNF-*α*, IL-5, IL-1*β*, M-CSF, and RANTES and inhibition of NF-*κ*B in lung, MMP-9-positive, and MMP-12-positive cells and increased TIMP-1 expression.	Asthma model, elastase model	[62, 88]
Kuwanon G (**31**)	Reduction of IL-4, IL-5, and IL-13 in the sera and BALF	Asthma model	[63]
Naringin (**37**)	Reduction of IL-4, IL-5, IL-13, and INF-*δ* levels	Asthma model	[70, 71]
Apigenin (**38**)	Reduction of eosinophil infiltration in lung tissue and IL-6, TNF-*α*, and IL-17A levels	Asthma model	[72]

*Extracts of plants*			

*Astragalus membranaceus*	Reduction of eosinophils and lymphocyte infiltration. Modulate Th1/2 immune balance and activates PPAR	Asthma model	[69]
*Boerhavia procumbens*	Reduction in the infiltration of eosinophils and lymphocytes in lungs	Asthma model	[65]
*Ocimum gratissimum*	Reduction in the infiltration of eosinophils and IL-4 expression	Asthma model	[66]
*Punica granatum *	Reduction of eosinophils and cytokines IL-1*β* and IL-5	Asthma model	[75]
*Siegesbeckia glabrescens *	Reduction of the expression of iNOS and COX-2, cytokine IL-4, IL-5, and IL-13	Asthma model	[78]
Herbal formula PM014	Reduction of neutrophils, macrophages, and lymphocytes in BALF, TNF-*α*, and IL-6 levels,	Elastase model	[89]
*Callicarpa japonica *	Reduction of neutrophil infiltration, cytokines IL-6 and TNF-*α*, and the oxidative stress	Cigarette smoke model	[90]
